# Susceptibility loci for metabolic syndrome and metabolic components identified in Han Chinese: a multi‐stage genome‐wide association study

**DOI:** 10.1111/jcmm.13042

**Published:** 2017-03-30

**Authors:** Yimin Zhu, Dandan Zhang, Dan Zhou, Zhenli Li, Zhiqiang Li, Le Fang, Min Yang, Zhongyan Shan, Hong Li, Jianhua Chen, Xianghai Zhou, Wei Ye, Senhai Yu, Huabin Li, Libin Cai, Chengguo Liu, Jie Zhang, Lixin Wang, Yaxin Lai, Liansheng Ruan, Zhanhang Sun, Shuai Zhang, Hao Wang, Yi Liu, Yuyang Xu, Jie Ling, Chunxiao Xu, Yan Zhang, Duo Lv, Zheping Yuan, Jing Zhang, Yingqi Zhang, Yongyong Shi, Maode Lai

**Affiliations:** ^1^ Department of Epidemiology & BiostatisticsZhejiang University School of Public HealthHangzhouZhejiangChina; ^2^ Department of PathologyZhejiang University School of MedicineHangzhouZhejiangChina; ^3^ Key Laboratory of Disease Proteomics of Zhejiang ProvinceHangzhouZhejiangChina; ^4^ Bio‐X InstitutesKey Laboratory for the Genetics of Developmental and Neuropsychiatric Disorders (Ministry of Education)Shanghai Jiao Tong UniversityShanghaiChina; ^5^ Institute of Social Cognitive and Behavioral SciencesShanghai Jiao Tong UniversityShanghaiChina; ^6^ Zhejiang Provincial Center for Disease Control and PreventionHangzhouZhejiangChina; ^7^ Department of Nutrition and Food SafetyZhejiang University School of Public HealthHangzhouChina; ^8^ The Endocrine Institute and Liaoning Provincial Key Laboratory of Endocrine DiseasesDepartment of Endocrinology and MetabolismThe First Hospital of China Medical UniversityShenyangLiaoningChina; ^9^ Department of EndocrinologySir Run Run Shaw Hospital Affiliated to School of MedicineZhejiang UniversityHangzhouZhejiangChina; ^10^ Department of Endocrinology and MetabolismPeking University People's HospitalBeijingChina; ^11^ Peking University Diabetes CenterBeijingChina; ^12^ Key Laboratory of Laboratory MedicineMinistry of Education of ChinaSchool of Laboratory Medicine and Life ScienceWenzhou Medical UniversityWenzhouZhejiangChina; ^13^ Daicun Town Community Health Service Center, Xiaoshan DistrictHangzhouZhejiangChina; ^14^ Xiaoshan District Sixth People's HospitalHangzhouZhejiangChina; ^15^ Xiaoshan District Third People's HospitalHangzhouZhejiangChina; ^16^ Putuo District People's HospitalZhoushanZhejiangChina

**Keywords:** metabolic syndrome, genome‐wide association study, single‐nucleotide polymorphism, secondary signal, gene–environment interaction

## Abstract

Metabolic syndrome (MetS), a cluster of metabolic disturbances that increase the risk for cardiovascular disease and diabetes, was because of genetic susceptibility and environmental risk factors. To identify the genetic variants associated with MetS and metabolic components, we conducted a genome‐wide association study followed by replications in totally 12,720 participants from the north, north‐eastern and eastern China. In combined analyses, independent of the top known signal at rs651821 on *APOA5,* we newly identified a secondary triglyceride‐associated signal at rs180326 on *BUD13* (*P*_combined_ = 2.4 × 10^−8^). Notably, by an integrated analysis of the genotypes and the serum levels of APOA5, BUD13 and triglyceride, we observed that BUD13 was another potential mediator, besides APOA5, of the association between rs651821 and serum triglyceride. rs671 (*ALDH2*), an east Asian‐specific common variant, was found to be associated with MetS (*P*_combined_ = 9.7 × 10^−22^) in Han Chinese. The effects of rs671 on metabolic components were more prominent in drinkers than in non‐drinkers. The replicated loci provided information on the genetic basis and mechanisms of MetS and metabolic components in Han Chinese.

## Introduction

Metabolic syndrome (MetS) is characterized by a cluster of metabolic disorders including obesity, dyslipidemia, elevated fasting plasma glucose and elevated blood pressure 1. MetS increases the risks for diabetes mellitus, cardiovascular diseases and cancers as well as increased mortality from all causes 2, 3, 4, 5. The prevalence of MetS (as defined by the International Diabetes Federation consensus in 2005) was reported to be 16.5% from a cross‐sectional study in Chinese adults aged 35–74 years in 2000–2001 6. The estimated prevalence had increased to 18.2% according to the China Health and Nutrition Survey in 2009 7. Given that the prevalence of MetS is high and increasing quickly in the Chinese population, strategies for its early detection and effective intervention are urgently needed.

Both environmental and genetic factors, as well as their interactions, contribute to the incidence of MetS. The heritability has been estimated to be up to 50% for some metabolic components and 13–30% for MetS 8, 9, 10, 11, 12. Efforts have been made to map the metabolic‐associated single‐nucleotide polymorphisms (SNPs) using a single component as the quantitative outcome 13, 14, 15, 16, 17, 18, 19. Consider MetS as a binary outcome, several genome‐wide association studies (GWAS) attempted to find susceptible genetic loci affecting multiple metabolic outcome 20, 21, 22, 23, 24. From these studies, tagSNPs mainly including rs9939609 on *FTO*, rs629301 on *SORT1*, rs12678919 near *LPL*, rs1532085 on *LIPC*, rs651821 on *APOA5*, rs7412 on *APOE*, rs1532624 on *CETP*, rs671 on *ALDH2*, rs4607517 on *GCK* and other variants had been reported as metabolic‐associated loci. Among these tagSNPs, some casual variants had been located at functional regions of *APOA5* (rs2266788, 3′UTR), *ALDH2* (rs671, exon) in further studies 25, 26. However, the known genetic variations only explain a small part of the heritability and many potential genetic biomarkers of susceptibility remain to be discovered 27, 28.

It is generally accepted that the current criteria of MetS are integrated assessment strategies with only a binary outcome rather than precise levels of metabolic components. Screening for genetic susceptibility loci using these simplified definitions is based on the common phenomenon of pleiotropy in which one gene or one variant affects multiple phenotypes. Pleiotropy has been reported in genetic variations to be associated with high‐density lipoprotein cholesterol (HDL‐C), triglyceride (TG) and low‐density lipoprotein cholesterol (LDL‐C) 29. A systematic review of pleiotropy from a broader viewpoint suggested that a large number of genes and SNPs show pleiotropic effects in common complex diseases and traits 30. These findings suggested that the combined analysis of metabolic components as a whole is a critical supplement to metabolic component‐specific screening.

In the current study, we searched for genetic susceptibility loci for MetS and metabolic components using a multistage GWAS and aimed to understand the mechanism behind the associations in Han Chinese.

## Materials and methods

### Participants

In the genome‐wide discovery stage, 998 participants with MetS and 996 healthy controls were recruited from a community‐based survey in 2010–2011 in Linpu town, Xiaoshan District, Hangzhou, Zhejiang Province, China. For replication, seven independent cohorts with a total of 5514 cases and 5464 healthy controls were recruited from north‐eastern China (Shenyang cohort), northern China (Beijing cohort) and eastern China (Hangzhou, Daicun, Wenzhou, Zhoushan, and Zhejiang cohorts) (see Table S1 for further details). All participants were of Han Chinese ethnicity. Individuals were excluded if they had metabolic‐related interventions or had cancer, or serious chronic liver, lung, heart or kidney disorders. The study protocol was approved by the Research Ethics Committee at the School of Medicine, Zhejiang University. Each participant gave informed consent.

### Anthropometric measurements and epidemiological investigation

Anthropometric indices (weight, height, waist circumference and hip circumference) and blood pressure were measured following standard protocols. Body mass index (BMI) was calculated as the bodyweight in kilograms divided by the square of the height in metres. Waist‐to‐hip ratio (WHR) was calculated as waist circumference divided by hip circumference in centimetres. Serum biochemical parameters including fasting blood glucose (FBG), TG, HDL‐C and LDL‐C were measured after overnight fasting. The serum levels of APOA5 and BUD13 were measured using enzyme‐linked immunosorbent assay (ELISA) kits from Cusabio (Wuhan, Hubei, China, code: CSB‐E11901h, CSB‐EL002885HU). Alcohol consumption (classified as non‐drinker, light drinker or heavy drinker) was assessed in face‐to‐face interviews.

### Phenotypes and definitions

MetS was defined according to the criteria of the Metabolic Syndrome Study Cooperation Group of the Chinese Diabetes Society (CDS2004) requiring the presence of three or more of the following: BMI ≥25 kg/m^2^; systolic/diastolic blood pressure (SBP/DBP) ≥ 140/90 mmHg; FBG ≥ 6.1 mmol/l; TG ≥ 1.7 mmol/l; HDL‐C < 0.9 mmol/l (men) or <1.0 mmol/l (women) 31. Healthy controls were free of the above metabolic disorders. BMI, WHR, FBG, TG, HDL‐C and LDL‐C were treated as continuous variables in analyses.

### Genotyping, imputation and quality controls

Genomic DNA was isolated from whole blood using a TACO automatic nucleic acid extraction apparatus (GeneReach Biotechnology Corp., Taichung, Taiwan). Genotyping of the GWAS samples was conducted using Illumina Human‐OmniExpress 760k chips (Illumina, San Diego, CA, USA) in the Bio‐X Center, Shanghai Jiao Tong University, according to the manufacturer's protocol. Nineteen randomly selected samples were genotyped repeatedly, and the results showed ~99.9% concordance with corresponding samples in the discovery stage. The case and control samples were distributed evenly in each plate. Negative controls (without DNA template) were included on every plate.

Systematic quality control was conducted in the discovery stage (Fig. S1). SNPs were excluded if (*i*) they did not map to autosomal chromosomes, (*ii*) they had a minor allele frequency (MAF) <0.05 in current samples, (*iii*) the distribution in controls deviated from the Hardy–Weinberg equilibrium (*P* < 1.0 × 10^−4^) or (*iv*) the call rate was <95%. Samples were excluded from analyses if they (*i*) had overall successful genotyping call rates <95%, (*ii*) were population outliers according to the smartPCA program from EIGENSOFT 32 or (*iii*) had probable relatives (PI_hat > 0.25). After the quality control procedure, a total of 862 participants with MetS, 880 healthy controls and 533,059 SNPs were included in the discovery stage analyses.

The post‐quality control GWAS data were used for imputation. We imputed ungenotyped SNPs *via* IMPUTE2 33 with the haplotype reference data of 1092 individuals from the 1000 Genomes Project Phase I Integrated Variant Set Release (v3, March 2012) in NCBI build 37 (hg19) coordinates. SNPs with info score quality estimates of <0.8 were excluded from analyses. Finally, 4,642,479 SNPs were used for fine mapping and SNP function prediction.

In replication stage I, genotyping was performed with SNPscan™ (Genesky Biotechnologies Inc., Shanghai, China). The TaqMan genotyping platform (ABI 7900HT Real Time PCR system, Applied Biosystems, Foster City, CA, USA) was used in replication stages II and III. To evaluate the accuracy of SNPscan and the TaqMan platform, an additional 5% of samples was genotyped by Sanger sequencing. Genotyping call rate control (more than 95%) and Hardy–Weinberg equilibrium control (*P* > 1.0 × 10^−3^) were implemented in the replication stages.

### Strategies for signal selection, replication and statistical analyses

We considered MetS as a binary outcome and performed the Cochran–Armitage trend test in a logistic regression model with age, gender and the first two principal components as covariates using PLINK 1.07 34 in the discovery stage. Signals with a *P‐*value <5.0 × 10^−5^ were selected for replication. Metabolic components [BMI, WHR, FBG, TG (log‐transformed), HDL‐C and LDL‐C] were also considered as quantitative outcome for screening. Multiple linear regressions were performed for quantitative variables with age, sex and the first two principal components as covariates. BMI was considered as an additional covariant for screening SNPs affecting FBG, TG, HDL‐C and LDL‐C. Component‐associated signals with a *P*‐value <1.0 × 10^−5^ in the discovery phase were selected for replication.

To prune candidate SNPs sharing the same potential biological effects, the conditional analysis was performed with any two candidate SNPs within ~1 Mb. We kept only one of the statistically significant SNP if the other SNP had a *P*‐value > 0.05 in conditional analyses. As a result, 39 SNPs with independent effects were selected (Fig. S2). Thirty‐two of the 39 SNPs were successfully designed in replication phase I. Analyses were conducted in the replication stages with age and gender as covariates using PLINK 1.07 (the same as in the discovery stage). Combined effects were calculated with meta‐analyses using Stata 12.0 (STATA Corp, College Station, TX, USA) for the SNPs with *P*‐values <0.05 in the replication stage and a consistent direction of effect with discovery stage. SNPs with a combined *P*‐value <5.0 × 10^−8^ were regarded as significant at the genome‐wide level. Replications II and III were conducted if the *P*‐value of an SNP was <0.05 while the combined *P*‐value did not reach genome‐wide significant level (5.0 × 10^−8^) (Fig. S2). Meta‐analyses were applied to combine the results from different cohorts and stages with fixed‐effect models.

Linear regressions were conducted for the associations among tagSNPs (in the additive model), lifestyle (alcohol consumption) and serum levels of APOA5, BUD13, TG and HDL‐C using SAS for Windows (version 9.2, SAS Institute Inc., Cary, NC, USA).

The manhattan plots and quantile–quantile (Q‐Q) plots were drawn using R package ‘gap’. The genetic inflation factors were calculated using PLINK 1.07. The significant genome‐wide regions were plotted using the online tool LocusZoom based on the ASN population in hg19 coordinates 35. SNP function predictions were conducted after imputation‐based fine mapping. The genetic architectures surrounding replicated SNPs were assessed using the ENCODE database from the UCSC genome browser 36.

## Results

After quality control, 1742 participants (862 MetS cases and 880 healthy controls) and 533,059 genotyped SNPs were included for analyses in the discovery stage. Replication samples consisted of 656 cases and 933 controls for phase I, 709 cases and 1921 controls for phase II, and 4149 cases and 2610 controls for phase III. The characteristics of these participants in each stage are shown in Table S2. The inflation factors ranged from 1.01 to 1.02 for MetS and metabolic components, suggesting little evidence of population stratification after quality control.

### Association analyses for MetS and metabolic components

**Figure 1:**
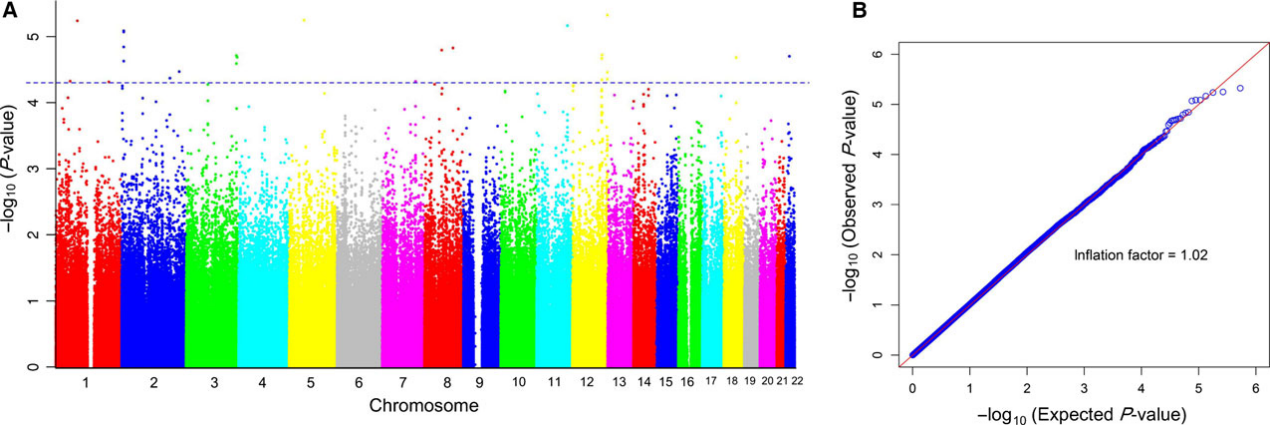
The manhattan (A) and quantile–quantile (Q‐Q) plots (B) of genome‐wide association study of metabolic syndrome. Manhattan and Q‐Q plots were constructed using the *P*‐values of SNPs that passed the quality control filters *via* logistic regression for metabolic syndrome adjusting for age, gender and the first two principal components in the genome‐wide discovery stage. Manhattan plot *Y*‐axis: −log_10_ (*P*‐value) of each SNP;* X*‐axis: chromosomes labelled with different colours. Q‐Q plot: observed (Y‐axis) *versus* expected (X‐axis) *P*‐values of SNPs; genetic inflation factor = 1.02.

To screen MetS‐associated variants, logistic regressions were performed for each SNP adjusted for age, gender and the first two principal components (Fig. S3) in the discovery stage. The genetic inflation factor was 1.02. The Q‐Q and manhattan plots are shown in Figure 1. Sixteen independent SNPs were found to be associated with MetS (*P* < 5.0 × 10^−5^) in the discovery stage. Then, these SNPs were genotyped in replication stage I. Two SNPs (rs651821 and rs671) were associated with MetS (*P* < 0.05, Table S3), and their effects were consistent with the results in the discovery stage. rs651821 and rs671 were further genotyped in replication stages (Table 1). The combined analyses presented that the C allele of rs651821 increases the risk of MetS with an odds ratio (OR) and 95% confidence interval (CI) of 1.28 (1.20, 1.36), with a combined *P* = 4.2 × 10^−17^. The A allele of rs671 decreased the risk of MetS with an OR and 95% CI of 0.71 (0.67, 0.76), combined *P* = 9.7 × 10^−22^. The effect sizes in each stage are presented in Table 1 and Table S3. Considering the age difference between cases and controls in some cohorts, we also classified the samples into three age groups (≤ 30, 31–60 and >60 years) and then performed age‐stratified analyses for the MetS‐associated SNPs. The effects of rs651821 and rs671 were stable among different age groups, and the results were consistent with the overall analyses (Table S4).

**Table 1:** SNPs associated with metabolic syndrome with genome‐wide significance in combined analyses

Phenotype	SNP	Chr	Position (bp)	Gene	Alleles^a^	MAF	Stages	*N*	OR (95% CI)	*P*‐value	*P*‐heterogeneity
MetS	rs651821	11	116 662 579	*APOA5*	C/T	0.28	Discovery	1742	1.30 (1.10, 1.49)	6.1 × 10^−06^	–
							Replication I	1580	1.31 (1.05, 1.56)	2.8 × 10^−04^	0.18
							Replication II	2494	1.27 (1.09, 1.46)	1.9 × 10^−04^	0.06
							Replication III	6113	1.27 (1.16, 1.37)	2.3 × 10^−08^	–
							Combined	11 929	1.28 (1.20, 1.36)	4.2 × 10^−17^	0.99
MetS	rs671	12	112 241 766	*ALDH2*	A/G	0.29	Discovery	1741	0.68 (0.59, 0.79)	1.0 × 10^−05^	–
							Replication I	1581	0.80 (0.63, 0.96)	2.9 × 10^−02^	0.96
							Replication II	2359	0.80 (0.67, 0.93)	5.5 × 10^−03^	0.56
							Replication III	6759	0.70 (0.64, 0.75)	1.3 × 10^−19^	–
							Combined	12 440	0.71 (0.67, 0.76)	5.4 × 10^−28^	0.29

*ALDH2*, aldehyde dehydrogenase 2; *APOA5*, apolipoprotein A‐V; Chr, chromosome; CI, confidence interval; MAF, minor allele frequency; N, number of participants; OR, odds ratio; MetS, metabolic syndrome; SNP, single‐nucleotide polymorphism. The OR, 95% CI, and *P*‐value of SNPs were estimated in the additive model by logistic regression for MetS adjusted for age, gender and the first two principal components.

a Alleles: minor allele/major allele; the minor allele was considered to be the effective allele.

In order to screen the SNPs associated with metabolic components, linear regressions were conducted using BMI, WHR, FBG, TG (log‐transformed), HDL‐C and LDL‐C as dependent variables. Q‐Q plots and manhattan plots are shown in Fig. S4. The genetic inflation factors ranged from 1.01 to 1.02 for these quantitative outcome. In the discovery stage, 24 SNPs had independent effects on the metabolic components with *P*‐value < 1.0 × 10^−5^ after conditional analyses. In replication stage I, rs1506525, rs4532958 and rs445925 were associated with BMI, WHR and LDL‐C (*P* < 0.05), respectively. A marginally significant association was found between rs180326 and TG after controlling for the top signal of rs651821 (*P* = 0.063). Combining the results of discovery and replication stage I, the association of rs445925 with LDL‐C reached significance at the genome‐wide level (*P*_combined_ = 1.1 × 10^−13^). The A allele of rs445925 was associated with a decreased level of LDL‐C [beta 95% CI = −0.22 (−0.28, −0.16)]. rs1506525, rs4532958 and rs180326 were further replicated as the combined *P*‐values did not reach the genome‐wide threshold for significance. We further genotyped rs651821 in replication stages II and III to determine whether the signal at rs180326 was independent of rs651821.

In replication stage II, rs180326 was associated with TG after controlling for the signal at rs651821 in replication stages II (*P* = 0.043) and III (*P* = 1.5 × 10^−5^). The combined effect size of the C allele of rs180326 and its 95% CI was −0.04 (−0.05, −0.03), *P*_combined_ = 2.4 × 10^−8^ in conditional analysis. For rs4532958, heterogeneity of the effect across stages was observed (*P*_heterogeneity_ < 0.05). The combined effect of rs4532958 (effect allele = C) was −0.008 (95% CI = −0.013, −0.004, *P*_combined_ = 5.1 × 10^−4^), estimated using random‐effect model. No significant effect of rs1506525 was found in replication stage II (Table S5).

### A novel secondary TG‐associated signal at rs180326 on *BUD13*

**Figure 2:**
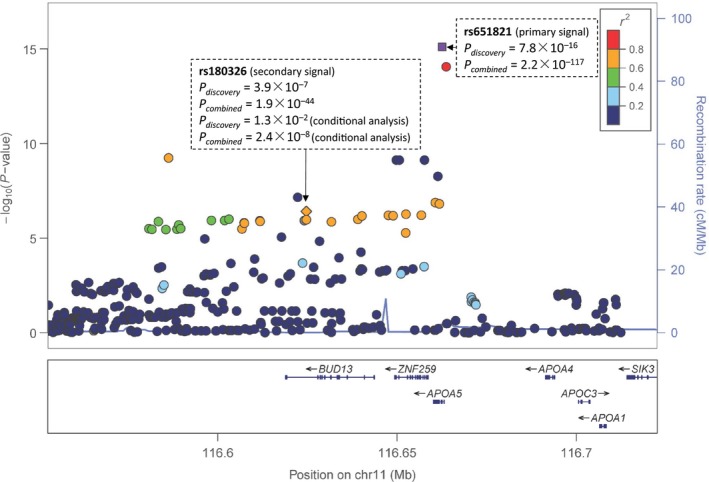
The regional plots of the top signal at rs651821 and the secondary signal at rs180326 for triglyceride. The regional plots were plotted *via* the online tool LocusZoom using ASN population as reference for LD calculations in hg19 coordinates. *P*‐values used for the regional plot were estimated from the discovery stage. The combined *P*‐values were given for the two signals. For rs180326, the conditional analysis was performed adjusting the top signal at rs651821.

The top knew TG‐associated signal of rs651821 was localized in the 5′UTR of *APOA5*, which belongs to the apolipoprotein gene cluster on chromosome 11q23 (Figure 2). A novel secondary TG‐associated signal at rs180326 on *BUD13* was replicated after controlling for the effect of rs651821. The combined effect size of the minor allele of rs180326 was −0.04 (95% CI: −0.05, −0.03 *P* = 2.4 × 10^−8^) with and 0.06 (95% CI: 0.05, 0.06 *P* = 1.9 × 10^−44^) without controlling for the top signal of rs651821. The opposite effects of rs180326 were stable in different stages (Table 2).

**Table 2:** SNPs associated with metabolic‐related phenotypes with genome‐wide significance in combined analyses

Phenotype	SNP	Chr	Position (bp)	Gene	Alleles*	MAF	Stages	*N*	Beta (95% CI)	*P*‐value	*P*‐heterogeneity
HDL‐C	rs651821	11	116 662 579	*APOA5*	C/T	0.28	Discovery	1740	−0.08 (−0.11, −0.05)	6.5 × 10^−07^	‐
							Replication I	1582	−0.05 (−0.07, −0.02)	2.9 × 10^−04^	0.38
							Replication II	2522	−0.09 (−0.11, −0.07)	3.3 × 10^−18^	0.77
							Replication III	6321	−0.06 (−0.07, −0.05)	5.0 × 10^−24^	‐
							Combined	12 165	−0.07 (−0.08, −0.06)	6.0 × 10^−48^	0.17
TG	rs651821	11	116 662 579	*APOA5*	C/T	0.28	Discovery	1740	0.08 (0.06, 0.10)	7.8 × 10^−16^	‐
							Replication I	1580	0.07 (0.05, 0.09)	1.9 × 10^−11^	0.09
							Replication II	2512	0.08 (0.07, 0.10)	2.2 × 10^−26^	0.44
							Replication III	6302	0.09 (0.08, 0.10)	4.9 × 10^−61^	‐
							Combined	12 134	0.08 (0.08, 0.09)	2.2 × 10^−117^	0.35
TG	rs180326	11	116 624 703	*BUD13*	C/A	0.22	Discovery	1740	0.05 (0.03, 0.07)	3.9 × 10^−07^	‐
								1740#	−0.04 (−0.08, −0.01)#	1.3 × 10^−02^ #	‐#
							Replication I	1575	0.05 (0.03, 0.07)	4.3 × 10^−06^	0.24
								1570#	−0.04 (−0.07, 0.00)#	6.3 × 10^−02^ #	0.97#
							Replication II	2507	0.06 (0.05, 0.08)	2.0 × 10^−13^	0.39
								2454#	−0.03 (−0.06, 0.00)#	4.3 × 10^−02^ #	0.30#
							Replication III	6348	0.06 (0.05, 0.07)	4.3 × 10^−24^	‐
								6057#	−0.04 (−0.06, −0.02)#	1.5 × 10^−05^ #	‐#
							Combined	12 170	0.06 (0.05, 0.06)	1.9 × 10^−44^	0.54
								11 821#	−0.04 (−0.05, −0.03)#	2.4 × 10^−08^ #	0.97#
LDL‐C	rs445925	19	45 415 640	*APOC1*	A/G	0.09	Discovery	1738	−0.27 (−0.35, −0.19)	4.1 × 10^−12^	‐
							Replication I	1524	−0.15 (−0.24, −0.06)	8.4 × 10^−04^	0.64
							Combined	3262	−0.22 (−0.28, −0.16)	1.1 × 10^−13^	0.05

*APOA5*: apolipoprotein A‐V; *APOC1*: apolipoprotein C‐I; *BUD13*: BUD13 homologue; Chr, chromosome; CI, confidence interval; N, number of participants; SNP, single‐nucleotide polymorphism. The Beta 95%CI, and *P*‐value of SNPs were estimated in the additive model by linear regression for each component adjusted for age, gender and the first two principal components. The serum level of TG was log‐transformed before analyses. For rs180326, regression models were used with (^#^) and without controlling for the top signal (rs651821) in this region.

*Alleles: minor allele/major allele; the minor allele was considered to be the effective allele.

**Figure 3:**
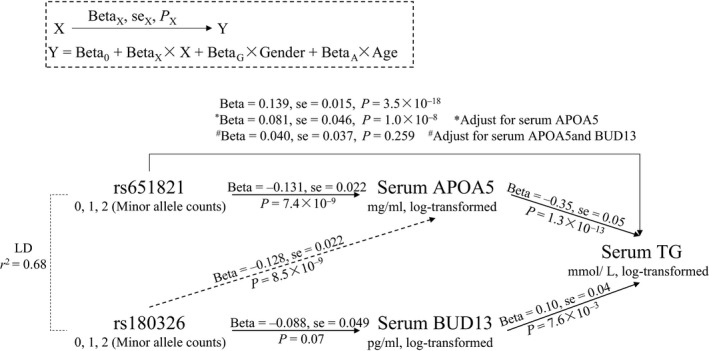
rs651821 and rs180326 were associated with serum levels of TG *via* variations in serum APOA5 and BUD13. Linear regression models were used to test the association between tagSNPs and serum level of APOA5, BUD13 and TG. An example is shown in the dashed box. We controlled for the serum levels of APOA5 and BUD13 in the model (* and ^#^) to test the association of rs651821 and TG.

To uncover the mechanism behind the opposite effects of the novel secondary signal, we measured the serum levels of APOA5 and BUD13 using ELISA kits as discribed in method. As shown in Figure 3, we observed that the minor allele C of rs651821 was associated with a lower level of APOA5 than the T allele (*P* = 7.4 × 10^−9^). The serum level of APOA5 was associated with TG (beta = −0.35, *P* = 3.3 × 10^−12^). The minor allele C of rs180326 was associated with a decreased serum level of BUD13 (*P* = 0.07). The serum level of BUD13 was associated with an increased level of serum TG (beta = 0.10, *P* = 7.6 × 10^−3^) and explained 14.2% of the serum TG variance. From the associations observed above, we speculated that the association between rs180326 and serum TG was masked by the LD between rs180326 and rs651821 before adjusting the top signal. Therefore, the combined effect of the minor allele C of rs180326 was inconsistent with and without controlling for the top signal at rs651821. Additionally, to determine whether APOA5 and BUD13 mediate the associations between rs651821 and metabolic components, we performed linear regressions and found that the association between rs651821 and TG was partly independent of the mediator APOA5 (*P* < 0.05 after controlling for the serum level of APOA5). No statistical association between rs651821 and TG was found when we added serum level of BUD13 as a covariate in the regression model (*P* = 0.259).

### Associations among rs671 (on *ALDH2*), alcohol consumption and MetS

rs671 is known to be a non‐synonymous mutation on *ALDH2*. As shown in Table S6, the minor allele frequency of rs671 is much higher in Asians (0.20), especially in the Han Chinese population (0.29) than in European (<0.01). Results showed that rs671 was strongly associated with alcohol consumption (*P*_combined_ = 1.7 × 10^−58^) in 4295 participants from the Beijing, Shenyang, Xiaoshan and Hangzhou cohorts. The interaction between the genotypes of rs671 and alcohol consumption status was found for MetS (*P* = 0.014). Stratification analysis showed that rs671 was significantly associated with MetS in drinkers (*P* = 7.5 × 10^−6^). Whereas only marginal significance in non‐drinkers (*P* = 0.097) was observed. Similar results were obtained in the associations with obesity and the other metabolic‐related components BMI, WHR, SBP and TG (Table 3). Then, we adjusted the alcohol consumption levels in drinkers in regression models. Our results suggested that these associations were partly independent of the alcohol consumption levels.

**Table 3:** Associations of rs671 and metabolic components were more evident in drinkers

Phenotypes	Drinking status	Phenotype levels by rs671 genotypes		*P*‐value^a^	*P*‐value^b^	*P*‐value^c^
		GG	GA or AA			
MetS (*N* case/control, case %)	Non‐drinker	224/880 (20.3%)	321/1388 (18.8%)	0.097		0.014
	Drinker	236/663 (26.3%)	89/494 (15.3%)	7.5 × 10^−6^	1.8 × 10^−4^	
BMI (kg/m^2^)	Non‐drinker	22.23 ± 3.22	22.20 ± 3.36	0.474		0.004
	Drinker	22.91 ± 3.28	22.13 ± 2.97	2.2 × 10^−5^	2.5 × 10^−5^	
WHR	Non‐drinker	0.84 ± 0.07	0.85 ± 0.07	0.851		8.0 × 10^−4^
	Drinker	0.88 ± 0.07	0.86 ± 0.06	8.3 × 10^−6^	2.0 × 10^−5^	
SBP (mmHg)	Non‐drinker	123.56 ± 20.25	124.49 ± 20.49	0.776		3.1 × 10^−4^
	Drinker	128.20 ± 21.05	122.61 ± 18.81	5.3 × 10^−6^	8.7 × 10^−6^	
DBP (mmHg)	Non‐drinker	72.72 ± 11.43	72.21 ± 12.19	0.049		0.161
	Drinker	75.44 ± 12.75	73.24 ± 12.58	0.002	0.003	
FBG (mmol/l)	Non‐drinker	4.91 ± 1.36	4.83 ± 1.20	0.041		0.017
	Drinker	4.96 ± 1.21	4.64 ± 0.90	8.7 × 10^−7^	1.4 × 10^−6^	
TG (mmol/l)	Non‐drinker	1.03 (0.75, 1.48)	1.07 (0.77, 1.48)	0.670		5.3 × 10^−4^
	Drinker	1.17 (0.85, 1.68)	1.05 (0.77, 1.43)	7.6 × 10^−6^	5.6 × 10^−6^	
HDL‐C (mmol/l)	Non‐drinker	1.37 ± 0.28	1.34 ± 0.28	0.140		0.500
	Drinker	1.44 ± 0.34	1.40 ± 0.30	0.011	0.023	
LDL‐C (mmol/l)	Non‐drinker	2.72 ± 0.78	2.77 ± 0.74	0.553		0.703
	Drinker	2.80 ± 0.76	2.79 ± 0.72	0.719	0.757	

BMI, body mass index; DBP, diastolic blood pressure; FBG, fasting blood glucose; HDL‐C, high‐density lipoprotein cholesterol; LDL‐C, low‐density lipoprotein cholesterol; MetS, metabolic syndrome; *N*, number of participants; SBP, systolic blood pressure; TC, total cholesterol; TG, triglyceride; WHR, waist‐to‐hip ratio.

The levels of obesity and metabolic components in the rs671 genotypes are showed as mean ± SD or median (25th percentile, 75th percentile) in drinkers and non‐drinkers.

a Logistic and linear regression for the association between rs671 and MetS and metabolic components in drinkers and non‐drinkers were conducted separately. Age, gender and study were set as covariants in regression models.

b Then, we adjusted for the alcohol consumption levels in drinkers.

c In addition, we tested the interaction between rs671 and alcohol consumption status for each trait.

## Discussion

In this multistage GWAS, using samples from north, north‐eastern and eastern China, we identified two SNPs (rs651821 on *APOA5* and rs671 on *ALDH2*) associated with MetS. Independent of the top signal at rs651821 in APOA cluster, rs180326 on BUD13 was observed as a novel secondary signal associated with serum TG.

The top TG‐associated signal was located at rs651821 on *APOA5*. This result was consistent with previous studies 37, 38, 39 as shown in Table S7. Recently, rs2266788, which was highly correlated with rs651821 (LD *r* ^2^ = 0.83), was demonstrated as a functional point mutation 25. In this study, it was proposed that the effect of rs2266788 (localized at the 3′UTR of *APOA5*) was mediated by the microRNA miR‐485‐5p expressed in the human liver. We performed conditional analyses in the discovery and replication stages and found a novel secondary signal at rs180326 on *BUD13* in this region. From the associations among rs651821, rs180326, serum levels of APOA5, BUD13 and TG, our results confirmed that the effect of rs180326 was masked by the strong LD between rs180326 and rs651821 before controlling the top signal in this region, which means that a false effect of rs180326 would be found without conditional analysis. Additionally, our results suggested that BUD13 was another potential mediator besides APOA5 for the association between the top signal at rs651821 and TG. Integrated with previous clues, the signal at rs651821 affects lipid metabolism *via* at least two causal variants. One of the causal mutations is the rs2266788 (on *APOA5*) which has been reported 25. Another causal variant (rs180326 or other loci) probably affects serum TG level *via* BUD13. *BUD13* is located in the APOC3/A4/A5 gene cluster on chromosome11q23.3. Genetic variants within this region are known to be associated with serum lipid components. A significant association between serum BUD13 and TG levels was observed in current study, whereas the role of BUD13 and the molecular mechanisms for these effects remained to be determined.

The non‐silence variant rs671 was localized in exon 12 of *ALDH2*, a key enzyme of alcohol metabolism. Mutation of rs671 from glutamate to lysine in *ALDH2* results in an enzyme that is rendered essentially inactive *in vivo* 26. Recent GWAS indicated that rs671 is associated with daily alcohol consumption 40, 41. Our results were consistent with the previous findings. The possible mechanism is that carriers of the A allele have a reduced capacity to catalyse acetaldehyde, and this leads to immediate and unpleasant symptoms, such as the flushing response and nausea, which probably result in reduced alcohol consumption. In addition, a significant interaction between alcohol consumption status and rs671 was found in the current study. In the stratified analysis of alcohol consumption status, a significant association between rs671 and MetS in drinkers was found. For drinkers, the A allele of rs671 was significantly associated with reduced risk of developing MetS. However, for non‐drinkers, there was no significant difference in the genotype frequency of rs671 between MetS and healthy controls. Furthermore, apart from influencing the alcohol consumptions, additional effects of rs671 were found for the associations between rs671 and metabolic components. Interactions between rs671 and alcohol consumption had been reported previously for serum TG 37 and other phenotypes including oesophageal cancer and an acute‐phase inflammation marker alpha‐1 antitrypsin 42, 43.

In conclusion, we performed a multiple‐stage GWAS for MetS and metabolic components in Han Chinese. A novel secondary TG‐associated signal at rs180326 on *BUD13* was replicated. rs651821 on *APOA5* was validated as a pleiotropic locus associated with MetS. In addition, evidence showing that, besides APOA5, BUD13 was another potential mediator for the association between rs651821 and serum TG. Interactions between rs671 and alcohol consumption status were found for MetS and metabolic components. The results of the current study provide novel evidence for the mechanisms underlying the development of MetS and metabolic components.

## Funding sources

This work was funded by National Key Technology R & D Program of China (2009BAI80B02, 2012BAI02B03); National Natural Science Foundation of China (81172755); The 111 Project (B13026); Program for Zhejiang Leading Team of Science and Technology Innovation (2010R50050); Zhejiang Provincial Program for the Cultivation of High‐level Innovative Health Talents.

## Author contributions

MD.L. organized this study. MD.L. and YM.Z. designed the study and supervised the epidemiological investigation. YY.S. supervised genotyping. YM.Z., DD.Z. and D.Z. performed the analyses and interpreted the main findings. YM.Z. and D.Z. drafted the manuscript with comments by all co‐authors. DD.Z., ZQ.L. JH.C. and ZL.L. supervised the raw data management and quality controls. L.F., M.Y., ZY.S., H.L., XH.Z., W.Y., SH.Y., HB.L., LB.C., CG.L., J.Z., LX.W., YX.L., LS.R. and ZH.S. conducted the recruitment of the samples and data collection. S.Z., H.W., YY.X., J.L., CX.X., Y.Z., D.L., ZP.Y., ZL.L., Y.L. and D.Z. contributed to DNA extraction and genotyping.

## Conflicts of interest

The authors declare that they have no competing interests.

## Supporting information


**Figure S1** Flow‐chart of the quality‐control procedure in the discovery phase.
**Figure S2** Flow‐chart of the study design.
**Figure S3** Principal component analysis showing minimal evidence of population stratification in discovery stage.
**Figure S4** Manhattan and Q‐Q plots for genome‐wide association studies of obesity and metabolic components (A) to (F).
**Table S1** The cohorts in the discovery and replication stages
**Table S2** Samples in the discovery and replication stages
**Table S3** SNPs associated with metabolic syndrome in the discovery phase and replicated in further phases if necessary based on the replication strategy
**Table S4** Age‐stratified analyses of SNPs associated with MetS and metabolic components
**Table S5** SNPs associated with obesity and metabolic components in the discovery phase and replicated in further phases if necessary based on the replication strategy
**Table S6** Global MAF of five SNPs associated with MetS and metabolic components

**Table S7** Results for the association analyses of MetS and metabolic conponents in the dataset of current study (discovery stage) for the previously reported SNPs.
