# Supplementary material for: Susceptibility loci for metabolic syndrome and metabolic components identified in Han Chinese: a multi‐stage genome‐wide association study
Source: J Cell Mol Med. 2017 Mar 30;21(6):1106–16. doi: 10.1111/jcmm.13042 (PMC5431133; doi:10.1111/jcmm.13042)
Supplement: Supplementary file 1 — Figure S1 Flow‐chart of the quality‐control procedure in the discovery phase. Figure S2 Flow‐chart of the study design. Figure S3 Principal component analysis showing minimal evidence of population stratification in discovery stage. Figure S4 Manhattan and Q‐Q plots for genome‐wide association studies of obesity and metabolic components (A) to (F). Table S1 The cohorts in the discovery and replication stages Table S2 Samples in the discovery and replication stages Table S3 SNPs associated with metabolic syndrome in the discovery phase and replicated in further phases if necessary based on the replication strategy Table S4 Age‐stratified analyses of SNPs associated with MetS and metabolic components Table S5 SNPs associated with obesity and metabolic components in the discovery phase and replicated in further phases if necessary based on the replication strategy Table S6 Global MAF of five SNPs associated with MetS and metabolic components [file JCMM-21-1106-s001.docx]

**Susceptibility loci for metabolic syndrome and metabolic components identified in Han Chinese: a multi-stage genome-wide association study**

**S1 Table.** The cohorts in the discovery and replication stages.

| **Cohort name** | **Stage** | **Sample size**  **(case / control)** | **Source** | **Date collected** |
| --- | --- | --- | --- | --- |
| Linpu | Discovery | 998 / 996 | Community-based cross-sectional study on metabolic syndrome in Linpu Town, Xiaoshan District, Hangzhou, Zhejiang Province | Jul, 2010 - Jul, 2011 |
| Shenyang | Replication I | 157 / 206 | Cross-sectional study in Shenyang, Liaoning Province | Mar - Oct, 2011 |
| Hangzhou | Replication I | 290 / 362 | Hospital-based survey from Sir Run Run Shaw Hospital, Jianggan District, Hangzhou, Zhejiang Province | Jun, 2010 - Oct, 2011 |
| Wenzhou | Replication I | 54 / 317 | Community-based survey in Wenzhou, Zhejiang Province | May - Jul, 2010 |
| Zhoushan | Replication I | 155 / 48 | Community-based survey in Zhoushan, Zhejiang Province | Jun - Jul, 2010 |
| Beijing | Replication II | 220 / 585 | Community-based survey in Pinggu District, Beijing | Mar - Nov, 2012 |
| Daicun | Replication II | 489 / 1336 | Community-based cross-sectional study on metabolic syndrome in Daicun Town, Xiaoshan District, Hangzhou, Zhejiang Province | Apr - Jul, 2012 |
| Zhejiang | Replication III | 4049 / 2610 | Cross-sectional study in Zhejiang Province on metabolic syndrome. Eleven cities (Ningbo, Wenzhou, Jinhua, Hangzhou, Qvzhou, Huzhou, Shaoxing, Jiaxing, Zhoushan, Lishui, and Taizhou) were included in this survey. | Jun - Oct, 2010 |

**S2 Table.** Samples in the discovery and replication stages.

| Stage |  | Discovery | |  | Replication I | | | | | | | |  | Replication stage II | | | |  | Replication stage III | |
| --- | --- | --- | --- | --- | --- | --- | --- | --- | --- | --- | --- | --- | --- | --- | --- | --- | --- | --- | --- | --- |
| Cohort |  | Linpu | |  | Shenyang | | Hangzhou | | Wenzhou | | Zhoushan cohort | |  | Beijing cohort | | Daicun cohort | |  | Zhejiang cohort | |
| Status |  | Case | Control |  | Case | Control | Case | Control | Case | Control | Case | Control |  | Case | Control | Case | Control |  | Case | Control |
| N |  | 880 | 862 |  | 157 | 206 | 290 | 362 | 54 | 317 | 155 | 48 |  | 220 | 585 | 489 | 1336 |  | 4049 | 2610 |
| Gender | Male | 458 | 448 |  | 109 | 76 | 74 | 118 | 14 | 102 | 67 | 20 |  | 121 | 255 | 189 | 636 |  | 1292 | 1211 |
|  | Female | 422 | 414 |  | 48 | 130 | 216 | 244 | 40 | 215 | 88 | 28 |  | 99 | 330 | 300 | 700 |  | 2757 | 1399 |
| Age | years | 59.9±10.7 | 55.3±12.0 |  | 54.1±7.6 | 51.1±7.2 | 59.74±6.24 | 53.58±6.56 | 60.44±9.21 | 56.22±11.40 | 56.09±12.53 | 50.92±15.63 |  | 55.1±8.7 | 52.8±8.7 | 59.42±11.3 | 49.87±16.05 |  | 54.8±12.8 | 50±12.6 |
| BMI | kg/m^2^ | 27.02±2.63 | 21.49±1.92 |  | 27.97±3.01 | 23.29±2.87 | 27.36±2.93 | 21.93±2.19 | 27.10±2.09 | 21.89±1.99 | 26.54±2.84 | 21.20±2.10 |  | 29.02±3.1 | 25.52±3.54 | 27.05±2.64 | 21.19±2.12 |  | 26.38±2.89 | 21.10±2.14 |
| WHR |  | 0.91±0.06 | 0.83±0.05 |  | 0.94±0.05 | 0.88±0.06 | 0.93±0.05 | 0.84±0.06 | 0.90±0.06 | 0.89±0.06 | 0.92±0.06 | 0.83±0.06 |  | 0.93±0.06 | 0.88±0.06 | 0.93±0.06 | 0.83±0.06 |  | 0.94±0.06 | 0.84±0.06 |
| SBP | mmHg | 158.01±17.18 | 120.48±11.31 |  | 142.95±15.13 | 117.48±15.04 | 149.88±16.26 | 114.09±12.77 | 140.11±18.74 | 112.00±12.99 | 142.78±20.70 | 117.69±12.43 |  | 145.05±18.15 | 127.76±15.78 | 154.24±15.55 | 117.95±12.24 |  | 138.26±19.91 | 116.55±12.75 |
| DBP | mmHg | 92.06±10.23 | 72.88±8.29 |  | 91.06±9.17 | 75.83±9.9 | 89.77±9.51 | 75.66±7.60 | 78.04±10.08 | 67.42±8.75 | 86.01±10.73 | 70.13±9.22 |  | 94.82±13.14 | 83.8±9.67 | 87.36±11.26 | 70.17±9.24 |  | 84.60±10.31 | 69.12±9.32 |
| FBG | mmol/L | 6.19±1.87 | 4.86±0.45 |  | 7.17±2.29 | 5.21±0.40 | 6.08±1.94 | 4.74±0.51 | 7.52±3.33 | 4.84±0.74 | 5.81±1.76 | 4.60±0.53 |  | 7.17±2.22 | 5.72±1.48 | 6.13±2.22 | 4.44±0.52 |  | 5.70±1.65 | 4.59±0.53 |
| TG | mmol/L | 2.49 (1.99, 3.45) | 1.17 (0.91, 1.39) |  | 2.30 (1.80, 3.30) | 1.10 (0.80, 1.50) | 2.51 (1.95, 3.63) | 1.03 (0.79, 1.29) | 2.34 (1.79, 3.55) | 0.96 (0.73, 1.26) | 0.77 (0.62, 1.38) | 1.81 (1.32, 2.48) |  | 1.93(1.45,2.68) | 1.09 (0.74, 1.55) | 2.47 (1.92, 3.34) | 0.97 (0.74, 1.24) |  | 1.86 (1.31, 2.59) | 0.93 (0.72, 1.23) |
| TC | mmol/L | 5.03±0.92 | 4.39±0.78 |  | 5.47±1.19 | 4.89±0.91 | 5.27±1.14 | 5.45±0.97 | 4.19±1.46 | 4.10±0.94 | 4.63±0.94 | 4.12±0.78 |  | 5.16±1.01 | 4.91±0.96 | 4.76±0.98 | 4.07±0.75 |  | 4.52±0.92 | 4.05±0.77 |
| HDL-C | mmol/L | 1.44±0.43 | 1.65±0.38 |  | 1.15±0.25 | 1.52±0.34 | 1.28±0.26 | 1.57±0.36 | 1.22±0.24 | 1.37±0.27 | 1.10±0.30 | 1.47±0.32 |  | 1.06±0.30 | 1.28±0.39 | 1.15±0.25 | 1.44±0.29 |  | 1.03±0.21 | 1.43±0.28 |
| LDL-C | mmol/L | 2.23±0.74 | 1.97±0.58 |  | 3.35±0.96 | 3.00±0.78 | 2.51±0.69 | 2.35±0.54 | - | 2.51±0.56 | 2.67±0.89 | 2.55±0.74 |  | 2.97±0.88 | 2.78±0.78 | 3.07±0.84 | 2.71±0.7 |  | 3.08±0.80 | 2.66±0.71 |

BMI, body mass index; DBP, diastolic blood pressure; FBG, fasting blood glucose; HDL-C, high-density lipoprotein cholesterol; LDL-C, low-density lipoprotein cholesterol; N, number of participants; SBP, systolic blood pressure; TC, total cholesterol; TG, triglyceride; WHR, waist-to-hip ratio.

Age and levels of BMI, WHR, SBP, DBP, FBG, TC, HDL-C, and LDL-C are presented as mean ± SD. Levels of TG are presented as median (25^th^ percentile, 75^th^ percentile).

**S1 Fig. Flow-chart of the quality-control procedure in the discovery phase.**


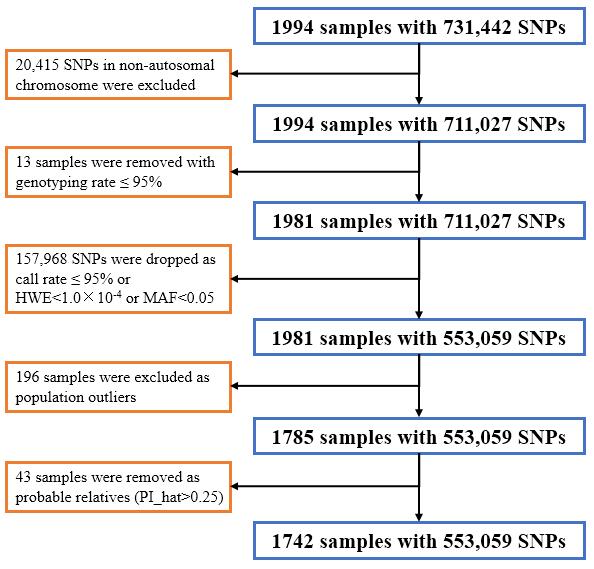


**S2 Fig. Flow-chart of the study design.** Obesity and the metabolic components BMI, WHR, FBG, TG (log-transformed), HDL-C, and LDL-C.


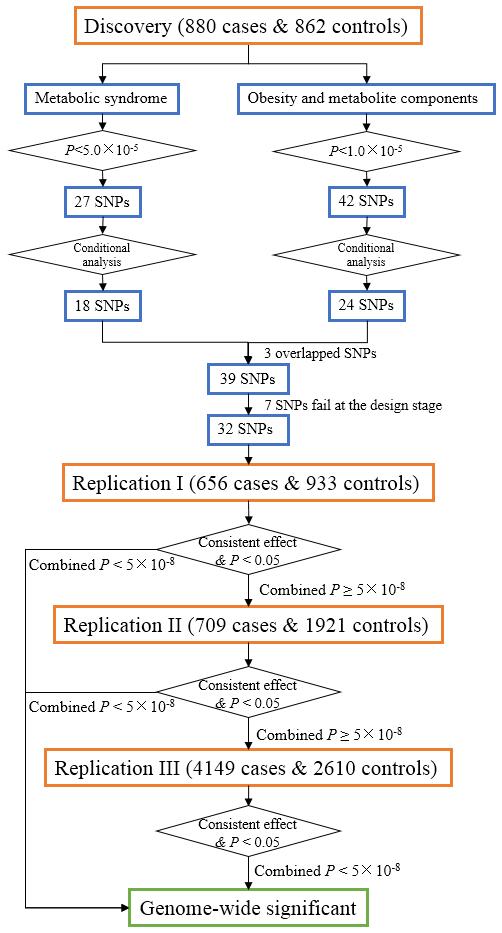


**S3 Fig. Principal component analysis showing minimal evidence of population stratification in discovery stage. The first two components (PC1 and PC2) derived from EIGENSTRAT are plotted for each participant.**


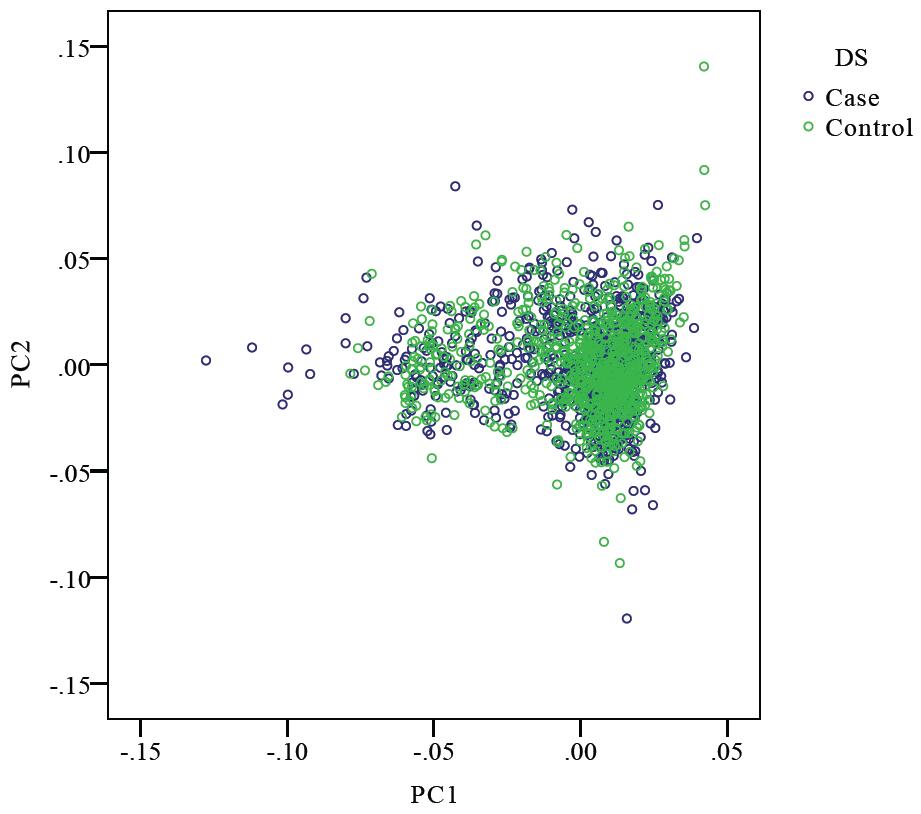


**S3 Table. SNPs associated with metabolic syndrome in the discovery phase and replicated in further phases if necessary based on the replication strategy.**

| Phenotype | SNP | Chr | Position (bp) | Gene | Alleles* | MAF | Stages | N | Beta (95%CI) | *P*-value |
| --- | --- | --- | --- | --- | --- | --- | --- | --- | --- | --- |
| MetS | rs10128884 | 12 | 132621624 | *DDX51* | A/G | 0.13 | Discovery | 1742 | 1.62 (1.32, 1.99) | 4.7×10^-06^ |
|  |  |  |  |  |  |  | Replication I | 1584 | 1.01 (0.82, 1.24) | 0.94 |
|  |  |  |  |  |  |  | Combined | 3326 | 1.27 (1.10, 1.47) | 1.1×10^-03^ |
|  | rs10217013 | 8 | 64878574 | *IFITM8P\|\|LOC100130155* | C/A | 0.39 | Discovery | 1740 | 0.73 (0.63, 0.84) | 1.6×10^-05^ |
|  |  |  |  |  |  |  | Replication I | 1583 | 1.09 (0.95, 1.26) | 0.24 |
|  |  |  |  |  |  |  | Combined | 3323 | 0.89 (0.81, 0.99) | 0.03 |
|  | rs11742315 | 5 | 55962593 | *LOC441073\|\|MAP3K1* | A/G | 0.15 | Discovery | 1742 | 0.64 (0.53, 0.78) | 5.6×10^-06^ |
|  |  |  |  |  |  |  | Replication I | 1586 | 1.11 (0.92, 1.34) | 0.28 |
|  |  |  |  |  |  |  | Combined | 3328 | 0.85 (0.74, 0.97) | 0.01 |
|  | rs11768153 | 7 | 125000368 | *LOC646837\|\|GRM8* | G/A | 0.28 | Discovery | 1742 | 0.73 (0.63, 0.85) | 4.8×10^-05^ |
|  |  |  |  |  |  |  | Replication I | 1581 | 1.13 (0.96, 1.33) | 0.14 |
|  |  |  |  |  |  |  | Combined | 3323 | 0.90 (0.80, 1.00) | 0.05 |
|  | rs16855489 | 2 | 217018256 | *XRCC5* | G/A | 0.27 | Discovery | 1737 | 1.39 (1.19, 1.63) | 3.4×10^-05^ |
|  |  |  |  |  |  |  | Replication I | 1582 | 0.99 (0.85, 1.16) | 0.9 |
|  |  |  |  |  |  |  | Combined | 3319 | 1.17 (1.05, 1.31) | 4.8×10^-03^ |
|  | rs16866569 | 2 | 8159830 | *LOC100129581\|\|C2orf46* | A/G | 0.07 | Discovery | 1742 | 0.55 (0.42, 0.71) | 8.1×10^-06^ |
|  |  |  |  |  |  |  | Replication I | 1584 | 0.87 (0.66, 1.15) | 0.32 |
|  |  |  |  |  |  |  | Combined | 3326 | 0.69 (0.57, 0.83) | 1.0×10^-04^ |
|  | rs2078851 | 12 | 111690579 | *CUX2* | G/A | 0.44 | Discovery | 1742 | 1.36 (1.18, 1.56) | 2.1×10^-05^ |
|  |  |  |  |  |  |  | Replication I | 1479 | - | - |
|  |  |  |  |  |  |  | Combined | 1742 | 1.36 (1.18, 1.56) | 2.1×10^-05^ |
|  | rs2510817 | 8 | 107486045 | *LOC100128259\|\|OXR1* | A/G | 0.25 | Discovery | 1709 | 0.70 (0.60, 0.82) | 1.5×10^-05^ |
|  |  |  |  |  |  |  | Replication I | 1584 | 0.93 (0.79, 1.08) | 0.34 |
|  |  |  |  |  |  |  | Combined | 3293 | 0.81 (0.72, 0.90) | 2.1×10^-04^ |
|  | rs671 | 12 | 112241766 | *ALDH2* | A/G | 0.29 | Discovery | 1741 | 0.68 (0.59, 0.79) | 1.0×10^-05^ |
|  |  |  |  |  |  |  | Replication I | 1581 | 0.80 (0.63, 0.96) | 0.03 |
|  |  |  |  |  |  |  | Replication II | 2359 | 0.80 (0.67, 0.93) | 5.5×10^-03^ |
|  |  |  |  |  |  |  | Replication III | 6759 | 0.70 (0.64, 0.75) | 1.3×10^-19^ |
|  |  |  |  |  |  |  | Combined | 12440 | 0.71 (0.67, 0.76) | 5.4×10^-28^ |
|  | rs651821 | 11 | 116662579 | *APOA5* | C/T | 0.28 | Discovery | 1742 | 1.30 (1.10, 1.49) | 6.1×10^-06^ |
|  |  |  |  |  |  |  | Replication I | 1580 | 1.31 (1.05, 1.56) | 2.8×10^-04^ |
|  |  |  |  |  |  |  | Replication II | 2494 | 1.27 (1.09, 1.46) | 1.9×10^-04^ |
|  |  |  |  |  |  |  | Replication III | 6113 | 1.27 (1.16, 1.37) | 2.3×10^-08^ |
|  |  |  |  |  |  |  | Combined | 11929 | 1.28 (1.20, 1.36) | 4.2×10^-17^ |
|  | rs6671173 | 1 | 55161844 | *C1orf175* | A/G | 0.26 | Discovery | 1742 | 1.37 (1.18, 1.60) | 4.7×10^-05^ |
|  |  |  |  |  |  |  | Replication I | 1584 | 1.11 (0.94, 1.30) | 0.22 |
|  |  |  |  |  |  |  | Combined | 3326 | 1.24 (1.11, 1.39) | 1.4×10^-04^ |
|  | rs6778567 | 3 | 192611340 | *C3orf59* | A/G | 0.21 | Discovery | 1742 | 1.46 (1.23, 1.73) | 2.0×10^-05^ |
|  |  |  |  |  |  |  | Replication I | 1565 | 0.81 (0.68, 0.97) | 0.02 |
|  |  |  |  |  |  |  | Combined | 3307 | 1.10 (0.97, 1.25) | 0.13 |
|  | rs742205 | 22 | 31223456 | *OSBP2* | A/G | 0.21 | Discovery | 1742 | 1.46 (1.23, 1.74) | 2.0×10^-05^ |
|  |  |  |  |  |  |  | Replication I | 1582 | 0.88 (0.74, 1.04) | 0.14 |
|  |  |  |  |  |  |  | Combined | 3324 | 1.13 (1.00, 1.28) | 0.05 |
|  | rs8082925 | 18 | 47269486 | *LOC100129143\|\|ACAA2* | G/A | 0.28 | Discovery | 1742 | 0.72 (0.62, 0.84) | 2.1×10^-05^ |
|  |  |  |  |  |  |  | Replication I | 1583 | 1.03 (0.88, 1.20) | 0.74 |
|  |  |  |  |  |  |  | Combined | 3325 | 0.85 (0.77, 0.95) | 4.6×10^-03^ |
|  | rs9782905 | 1 | 82219539 | *LOC729828\|\|LPHN2* | G/A | 0.15 | Discovery | 1742 | 0.63 (0.52, 0.77) | 5.8×10^-06^ |
|  |  |  |  |  |  |  | Replication I | 1584 | 0.97 (0.80, 1.16) | 0.71 |
|  |  |  |  |  |  |  | Combined | 3326 | 0.79 (0.69, 0.91) | 6.8×10^-04^ |
|  | rs998135 | 1 | 201075796 | *CACNA1S* | A/G | 0.23 | Discovery | 1741 | 0.72 (0.61, 0.84) | 4.9×10^-05^ |
|  |  |  |  |  |  |  | Replication I | 1581 | 1.10 (0.93, 1.30) | 0.26 |
|  |  |  |  |  |  |  | Combined | 3322 | 0.88 (0.79, 0.99) | 0.03 |

Chr, chromosome; CI, confidence interval; MAF, minor allele frequency; MetS, metabolic syndrome; N, number of participants; OR, odds ratio; SNP, single-nucleotide polymorphism.

The OR, 95%CI, and *P*-value of SNPs were estimated in the additive model by logistic regression for metabolic syndrome adjusted for age, gender, and the first two principal components.

*Alleles: minor allele/major allele; the minor allele was considered to be the effective allele.

**S4 Table. Age-stratified analyses of SNPs associated with MetS and metabolic components**

| Phenotype | SNP | Chr | Position (bp) | Gene | Alleles* | MAF | Age groups (years) | N | OR/beta (95%CI) | *P*-value |
| --- | --- | --- | --- | --- | --- | --- | --- | --- | --- | --- |
| MetS | rs651821 | 11 | 116662579 | *APOA5* | C/T | 0.28 | ≤30 | 764 | 1.59 (1.21, 2.09) | 9.30×10^-04^ |
|  |  |  |  |  |  |  | 31 - 60 | 7350 | 1.34 (1.24, 1.44) | 3.35×10^-13^ |
|  |  |  |  |  |  |  | >60 | 3815 | 1.26 (1.13, 1.40) | 4.59×10^-05^ |
|  |  |  |  |  |  |  | Combined | 11929 | 1.32 (1.24, 1.41) | 1.05×10^-18^ |
|  |  |  |  |  |  |  |  |  |  |  |
| MetS | rs671 | 12 | 112241766 | *ALDH2* | A/G | 0.29 | ≤30 | 828 | 0.73 (0.55, 0.97) | 2.71×10^-02^ |
|  |  |  |  |  |  |  | 31 - 60 | 7593 | 0.73 (0.68, 0.79) | 4.74×10^-15^ |
|  |  |  |  |  |  |  | >60 | 4019 | 0.71 (0.64, 0.79) | 3.30×10^-10^ |
|  |  |  |  |  |  |  | Combined | 12440 | 0.73 (0.68, 0.77) | 9.19×10^-25^ |

Chr, chromosome; CI, confidence interval; N, number of participants; SNP, single-nucleotide polymorphism.

*Alleles: minor allele/major allele; the minor allele was considered to be the effective allele.

The effect sizes and *P*-values of SNPs were combined from multiple stages in specific age groups.

The effect sizes and *P*-values of SNPs were estimated in the additive model by logistic regression for each component adjusted for age, gender, and the first two principal components.

The combined analyses were performed by meta-analyses using the results of age-stratified analyses.

**S4 Fig. Manhattan and Q-Q plots for genome-wide association studies of obesity and metabolic components (A) to (F).**

**
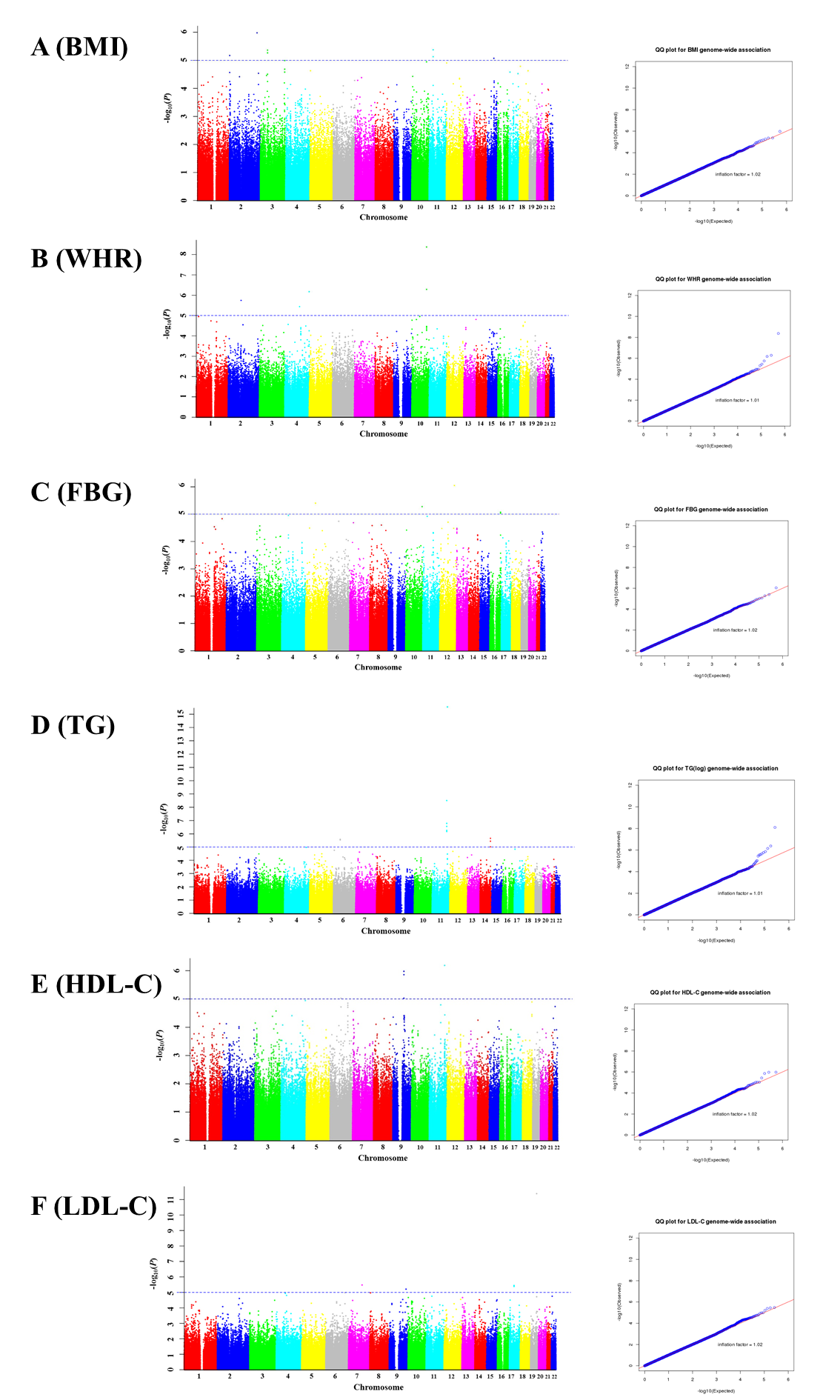
**

**S5 Table. SNPs associated with obesity and metabolic components in the discovery phase and replicated in further phases if necessary based on the replication strategy.**

| Phenotype | SNP | Chr | Position (bp) | Gene | Alleles* | MAF | Stages | N | Beta (95%CI) | *P*-value |
| --- | --- | --- | --- | --- | --- | --- | --- | --- | --- | --- |
| BMI | rs10200599 | 2 | 2838270 | *MYT1L\|\|LOC729897* | A/G | 0.40 | Discovery | 1738 | -0.55 (-0.79, -0.31) | 6.7×10^-06^ |
|  |  |  |  |  |  |  | Replication I | 1556 | -0.15 (-0.39, 0.09) | 0.23 |
|  |  |  |  |  |  |  | Combined | 3294 | -0.35 (-0.52, -0.18) | 5.2×10^-05^ |
|  | rs1506525 | 11 | 32044946 | *LOC729605\|\|RCN1* | G/A | 0.33 | Discovery | 1739 | 0.57 (0.32, 0.82) | 7.4×10^-06^ |
|  |  |  |  |  |  |  | Replication I | 1580 | 0.29 (0.04, 0.54) | 0.03 |
|  |  |  |  |  |  |  | Replication II | 2496 | 0.19 (-0.03, 0.41) | 0.09 |
|  |  |  |  |  |  |  | Combined | 5815 | 0.34 (0.20, 0.47) | 1.8×10^-06^ |
|  | rs16855489 | 2 | 217018256 | *XRCC5* | G/A | 0.27 | Discovery | 1734 | 0.68 (0.41, 0.95) | 1.1×10^-06^ |
|  |  |  |  |  |  |  | Replication I | 1581 | -0.05 (-0.32, 0.21) | 0.69 |
|  |  |  |  |  |  |  | Combined | 3315 | 0.31 (0.12, 0.50) | 1.6×10^-03^ |
|  | rs8025170 | 15 | 75170983 | *SCAMP2\|\|MPI* | G/A | 0.36 | Discovery | 1738 | -0.56 (-0.81, -0.32) | 8.5×10^-06^ |
|  |  |  |  |  |  |  | Replication I | 1583 | 0.04 (-0.21, 0.29) | 0.73 |
|  |  |  |  |  |  |  | Combined | 3321 | -0.26 (-0.44, -0.09) | 3.3×10^-03^ |
|  | rs9882932 | 3 | 56518184 | *C3orf51\|\|CCDC66* | A/G | 0.06 | Discovery | 1738 | 1.12 (0.64, 1.59) | 4.3×10^-06^ |
|  |  |  |  |  |  |  | Replication I | 1583 | 0.27 (-0.20, 0.75) | 0.25 |
|  |  |  |  |  |  |  | Combined | 3321 | 0.69 (0.36, 1.03) | 5.1×10^-05^ |
| WHR | rs4532958 | 10 | 115214154 | *TCF7L2 \|\|HABP2* | C/A | 0.33 | Discovery | 1640 | -0.013 (-0.017, -0.009) | 4.3×10^-09^ |
|  |  |  |  |  |  |  | Replication I | 1513 | -0.006 (-0.011, -0.001) | 0.012 |
|  |  |  |  |  |  |  | Replication II | 2490 | -0.006 (-0.010, -0.002) | 1.9×10^-03^ |
|  |  |  |  |  |  |  | Combined | 5643 | -0.008 (-0.013, -0.004) | 5.1×10^-04^ |
| FBG | rs11068350 | 12 | 117566820 | *TESC\|\|FBXO21* | G/A | 0.39 | Discovery | 1685 | -0.25 (-0.35, -0.15) | 9.0×10^-07^ |
|  |  |  |  |  |  |  | Replication I | 1530 | -0.01 (-0.12, 0.10) | 0.92 |
|  |  |  |  |  |  |  | Combined | 3215 | -0.14 (-0.21, -0.07) | 1.9×10^-04^ |
|  | rs12355487 | 10 | 130844717 | *LOC728327\|\|MGMT* | A/G | 0.23 | Discovery | 1739 | 0.27 (0.15, 0.38) | 5.3×10^-06^ |
|  |  |  |  |  |  |  | Replication I | 1435 | - | - |
|  |  |  |  |  |  |  | Combined | 1739 | 0.27 (0.15, 0.38) | 5.3×10^-06^ |
|  | rs12600202 | 16 | 84997875 | *LOC123862\|\|ZDHHC7* | A/G | 0.23 | Discovery | 1738 | 0.26 (0.15, 0.38) | 8.4×10^-06^ |
|  |  |  |  |  |  |  | Replication I | 1583 | -0.05 (-0.18, 0.08) | 0.46 |
|  |  |  |  |  |  |  | Combined | 3321 | 0.13 (0.04, 0.22) | 3.8×10^-03^ |
|  | rs4704624 | 5 | 79480620 | *SERINC5* | A/G | 0.18 | Discovery | 1739 | 0.29 (0.17, 0.42) | 3.9×10^-06^ |
|  |  |  |  |  |  |  | Replication I | 1587 | 0.11 (-0.03, 0.25) | 0.12 |
|  |  |  |  |  |  |  | Combined | 3326 | 0.21 (0.12, 0.31) | 7.1×10^-06^ |
| TG | rs651821 | 11 | 116662579 | *APOA5* | C/T | 0.28 | Discovery | 1740 | 0.08 (0.06, 0.10) | 7.8×10^-16^ |
|  |  |  |  |  |  |  | Replication I | 1580 | 0.07 (0.05, 0.09) | 1.9×10^-11^ |
|  |  |  |  |  |  |  | Replication II | 2512 | 0.08 (0.07, 0.10) | 2.2×10^-26^ |
|  |  |  |  |  |  |  | Replication III | 6302 | 0.09 (0.08, 0.10) | 4.9×10^-61^ |
|  |  |  |  |  |  |  | Combined | 12134 | 0.08 (0.08, 0.09) | 2.2×10^-117^ |
|  | rs180326 | 11 | 116624703 | *BUD13* | C/A | 0.22 | Discovery | 1740 | 0.05 (0.03, 0.07) | 3.9×10^-07^ |
|  |  |  |  |  |  |  |  | 1740# | -0.04 (-0.08, -0.01) # | 1.3×10^-02^# |
|  |  |  |  |  |  |  | Replication I | 1575 | 0.05 (0.03, 0.07) | 4.3×10^-06^ |
|  |  |  |  |  |  |  |  | 1570# | -0.04 (-0.07, 0.00) # | 6.3×10^-02^# |
|  |  |  |  |  |  |  | Replication II | 2507 | 0.06 (0.05, 0.08) | 2.0×10^-13^ |
|  |  |  |  |  |  |  |  | 2454# | -0.03 (-0.06, 0.00) # | 4.3×10^-02^# |
|  |  |  |  |  |  |  | Replication III | 6348 | 0.06 (0.05, 0.07) | 4.3×10^-24^ |
|  |  |  |  |  |  |  |  | 6057# | -0.04 (-0.06, -0.02) # | 1.5×10^-05^# |
|  |  |  |  |  |  |  | Combined | 12170 | 0.06 (0.05, 0.06) | 1.9×10^-44^ |
|  |  |  |  |  |  |  |  | 11821# | -0.04 (-0.05, -0.03) # | 2.4×10^-08^# |
| HDL-C | rs209411 | 6 | 83541333 | *TPBG\|\|UBE2CBP* | A/C | 0.11 | Discovery | 1734 | -0.10 (-0.14, -0.06) | 9.5×10^-06^ |
|  |  |  |  |  |  |  | Replication I | 1587 | -0.01 (-0.04, 0.03) | 0.72 |
|  |  |  |  |  |  |  | Combined | 3321 | -0.04 (-0.07, -0.01) | 3.4×10^-03^ |
|  | rs7864030 | 9 | 84369450 | *TLE1\|\|FLJ43950* | A/G | 0.47 | Discovery | 1738 | -0.07 (-0.10, -0.04) | 1.0×10^-06^ |
|  |  |  |  |  |  |  | Replication I | 1586 | 0.00 (-0.02, 0.02) | 0.88 |
|  |  |  |  |  |  |  | Combined | 3324 | -0.02 (-0.04, -0.01) | 5.8×10^-03^ |
|  | rs651821 | 11 | 116662579 | *APOA5* | C/T | 0.28 | Discovery | 1740 | -0.08 (-0.11, -0.05) | 6.5×10^-07^ |
|  |  |  |  |  |  |  | Replication I | 1582 | -0.05 (-0.07, -0.02) | 2.9×10^-04^ |
|  |  |  |  |  |  |  | Replication II | 2522 | -0.09 (-0.11, -0.07) | 3.3×10^-18^ |
|  |  |  |  |  |  |  | Replication III | 6321 | -0.06 (-0.07, -0.05) | 5.0×10^-24^ |
|  |  |  |  |  |  |  | Combined | 12165 | -0.07 (-0.08, -0.06) | 6.0×10^-48^ |
| LDL-C | rs10985976 | 9 | 126113594 | *STRBP\|\|CRB2* | G/A | 0.49 | Discovery | 1738 | -0.10 (-0.14, -0.06) | 6.2×10^-06^ |
|  |  |  |  |  |  |  | Replication I | 1329 | - | - |
|  |  |  |  |  |  |  | Combined | 1738 | -0.10 (-0.14, -0.06) | 6.2×10^-06^ |
|  | rs12602787 | 17 | 34882186 | *MYO19* | G/A | 0.10 | Discovery | 1738 | -0.18 (-0.25, -0.10) | 3.6×10^-06^ |
|  |  |  |  |  |  |  | Replication I | 1484 | 0.01 (-0.20, 0.22) | 0.93 |
|  |  |  |  |  |  |  | Combined | 3222 | -0.16 (-0.23, -0.09) | 1.5×10^-05^ |
|  | rs420437 | 7 | 101819315 | *CUX1* | A/G | 0.06 | Discovery | 1736 | -0.22 (-0.32, -0.13) | 3.3×10^-06^ |
|  |  |  |  |  |  |  | Replication I | 1491 | -0.06 (-0.18, 0.07) | 0.37 |
|  |  |  |  |  |  |  | Combined | 3227 | -0.16 (-0.23, -0.09) | 2.2×10^-05^ |
|  | rs445925 | 19 | 45415640 | *APOC1* | A/G | 0.09 | Discovery | 1738 | -0.27 (-0.35, -0.19) | 4.1×10^-12^ |
|  |  |  |  |  |  |  | Replication I | 1524 | -0.15 (-0.24, -0.06) | 8.4×10^-04^ |
|  |  |  |  |  |  |  | Combined | 3262 | -0.22 (-0.28, -0.16) | 1.1×10^-13^ |

Chr, chromosome; CI, confidence interval; HDL-C, high-density lipoprotein cholesterol; LDL-C, low-density lipoprotein cholesterol; N, number of participants; SNP, single-nucleotide polymorphism; TG, triglyceride; WHR, waist-to-hip ratio.

*Alleles: minor allele/major allele; the minor allele was considered to be the effective allele.

The Beta, 95%CI, and P-value of SNPs were estimated in the additive model by linear regression for each component adjusted for age, gender, and the first two principal components. For TG, HDL-C, and LDL-C, BMI was also adjusted as a covariant. The serum level of TG was log-transformed before analyses. For rs180326, regression models were used with (#) and without controlling for the top signal (rs651821) in this region.


**S6 Table. Global MAF of five SNPs associated with MetS and metabolic components.**

| Chromosome | Position  (hg19) | SNP | Global minor allele | Global major allele | Allele frequency (Global minor allele) | | | | | |
| --- | --- | --- | --- | --- | --- | --- | --- | --- | --- | --- |
|  |  |  |  |  | Global | African | Asian | European | African American | Current study |
| 10 | 115214154 | rs4532958 | C | A | 0.32 | 0.18 | 0.36 | 0.39 | 0.22 | 0.33 |
| 11 | 116624703 | rs180326 | G | T | 0.41 | 0.66 | 0.30 | 0.34 | 0.63 | 0.22 |
| 11 | 116662579 | rs651821 | C | T | 0.18 | 0.18 | 0.28 | 0.02 | 0.02 | 0.28 |
| 12 | 112241766 | rs671 | A | G | 0.04 | <0.01 | 0.20 | <0.01 | <0.01 | 0.29 |
| 19 | 45415640 | rs445925 | A | G | 0.15 | 0.32 | 0.06 | 0.13 | - | 0.09 |
